# A Novel Method for Measuring Serum Unbound Bilirubin Levels Using Glucose Oxidase–Peroxidase and Bilirubin-Inducible Fluorescent Protein (UnaG): No Influence of Direct Bilirubin

**DOI:** 10.3390/ijms21186778

**Published:** 2020-09-16

**Authors:** Sota Iwatani, Keiji Yamana, Hajime Nakamura, Kosuke Nishida, Takeshi Morisawa, Masami Mizobuchi, Kayo Osawa, Kazumoto Iijima, Ichiro Morioka

**Affiliations:** 1 Department of Neonatology, Hyogo Prefectural Kobe Children’s Hospital Perinatal Center, Kobe 650-0047, Japan; stiwatani_kch@hp.pref.hyogo.jp; 2 Department of Pediatrics, Kakogawa City Hospital, Kakogawa 675-8511, Japan; k-yamana@kjf.biglobe.ne.jp (K.Y.); m1917zou@gmail.com (T.M.); 3 Department of Pediatrics, Kobe University Graduate School of Medicine, Kobe 657-8501, Japan; nakamura.kch0201@gmail.com (H.N.); xitian1410@yahoo.co.jp (K.N.); iijima@med.kobe-u.ac.jp (K.I.); 4 Department of Developmental Pediatrics, Shizuoka Prefectural Shizuoka Children’s Hospital, Shizuoka 420-8660, Japan; mizobuchim@gmail.com; 5 Department of Medical Technology, Kobe Tokiwa University, Kobe 653-0838, Japan; k-ohsawa@kobe-tokiwa.ac.jp; 6 Department of Pediatrics and Child Health, Nihon University School of Medicine, Tokyo 173-8610, Japan

**Keywords:** albumin, analyzer, bilirubin encephalopathy, glucose oxidase-peroxidase method, indirect bilirubin, neonatal intensive care unit

## Abstract

The glucose oxidase–peroxidase (GOD–POD) method used to measure serum unbound bilirubin (UB) suffers from direct bilirubin (DB) interference. Using a bilirubin-inducible fluorescent protein from eel muscle (UnaG), a novel GOD–POD–UnaG method for measuring UB was developed. Newborn sera with an indirect bilirubin/albumin (iDB/A) molar ratio of <0.5 were classified into four groups of DB/total serum bilirubin (TB) ratios (<5%, 5–10%, 10–20%, and ≥20%), and the correlation between the UB levels and iDB/A ratio was examined. Linear regression analysis was performed to compare UB values from both methods with the iDB/A ratio from 38 sera samples with DB/TB ratio <5% and 11 samples with DB/TB ratio ≥5%. The correlation coefficient (*r*) between UB values and the iDB/A ratio for the GOD–POD method was 0.8096 (DB/TB ratio <5%, *n* = 239), 0.7265 (5–10%, *n* = 29), 0.7165 (10–20%, *n* = 17), and 0.4816 (≥20%, *n* = 16). UB values using the GOD–POD–UnaG method highly correlated with the iDB/A ratio in both <5% and ≥5% DB/TB ratio sera (*r* = 0.887 and 0.806, respectively), whereas a low correlation (*r* = 0.428) occurred for ≥5% DB/TB ratio sera using the GOD–POD method. Our GOD–POD–UnaG method can measure UB levels regardless of the presence of DB.

## 1. Introduction

An increase in serum unconjugated bilirubin (indirect bilirubin, iDB) levels, most importantly free bilirubin not bound to albumin (unbound bilirubin, UB), is associated with the development of serious brain injury in newborns, called bilirubin encephalopathy [1,2]. In Japan, bilirubin encephalopathy is an etiology of dyskinetic cerebral palsy and abnormal auditory brainstem response. This is of particular concern for extremely preterm infants [3,4], of which the prevalence is at least two per 1000 live births less than 30 weeks of gestational age [5]. Furthermore, children with bilirubin encephalopathy suffer from serious challenges in their daily lives [3,4].

An automated analyzer designed to measure UB serum/plasma levels was developed in 1982 (UB-Analyzer; UA-2, Arrows Co., Ltd., Osaka, Japan) using the glucose oxidase and peroxidase (GOD–POD) method [6,7]. To date, this is the only method approved for clinical use by the US Food and Drug Administration and the Ministry of Health, Labor, and Welfare in Japan. In this method using absorbance spectroscopy, albumin-unbound bilirubin is oxidatively degraded and readily converted into a colorless substance, whereas albumin-bound bilirubin tends not to be oxidatively degraded. Therefore, UB levels are calculated from the initial rate of oxidative degradation [6,7]. More specifically, UB levels are determined by colorimetrically monitoring the rate of decreasing absorbance associated with bilirubin pigments [1,6]. However, several factors may interfere with UB measurements using the GOD–POD method, resulting in the underestimation (from either sample dilution, ZE-bilirubin, or vitamin C) or overestimation (from conjugated bilirubin (direct bilirubin, DB), EZ-cyclobilirubin, or hemoglobin) [1]. Most importantly, DB affects the accuracy of UB measurement because DB is also easily changed into a colorless compound by POD. Consequently, this can lead to misjudgments and wrong decisions in clinical practice when planning treatments. The UB values displayed by the UB-Analyzer using the GOD–POD method can register higher than is actually present when the DB/TB ratio is high; a DB/TB ratio >10% overestimates UB levels compared to a ratio <10%, although the specific effect of DB on UB measurements by the GOD–POD method remains unclear [1]. This problem is more significant for newborn care in the neonatal intensive care unit (NICU) because >20% of hospitalized infants in Japanese NICUs have conjugated hyperbilirubinemia, which is especially prevalent among extremely preterm infants, small-for-gestational infants, and infants with gastrointestinal anomalies or chromosomal disorders [8].

In 2013, a bilirubin-inducible fluorescent protein from eel muscle (UnaG) was cloned, which is characterized as having high affinity and specific binding to iDB rather than DB [9]. In newborn sera, we confirmed the measurement of iDB directly using UnaG regardless of the presence of DB, bilirubin photoisomers, hemoglobin, or lipid emulsions [10]. To overcome the challenges of the GOD–POD method, we recently developed a novel method for measuring UB levels using GOD–POD in conjunction with UnaG, referred to as the GOD–POD–UnaG method, which was registered with the patent office on 12 June 2020 (registration number: 6716108).

The aims of our study involve further delineating the strengths of the GOD–POD–UnaG method with respect to the established GOD–POD method. First, we studied clinical serum samples to determine how much the DB/TB ratio would affect UB values when using the GOD–POD method (Study A). Next, we tested whether the GOD–POD–UnaG method could be used for UB measurements in newborn sera, regardless of those with a high DB/TB ratio (Study B).

## 2. Results

### 2.1. Study A: Impact of Serum DB Levels on the Correlations between iDB/A Ratio and Serum UB Levels in Clinical Data

**Figure 1:**
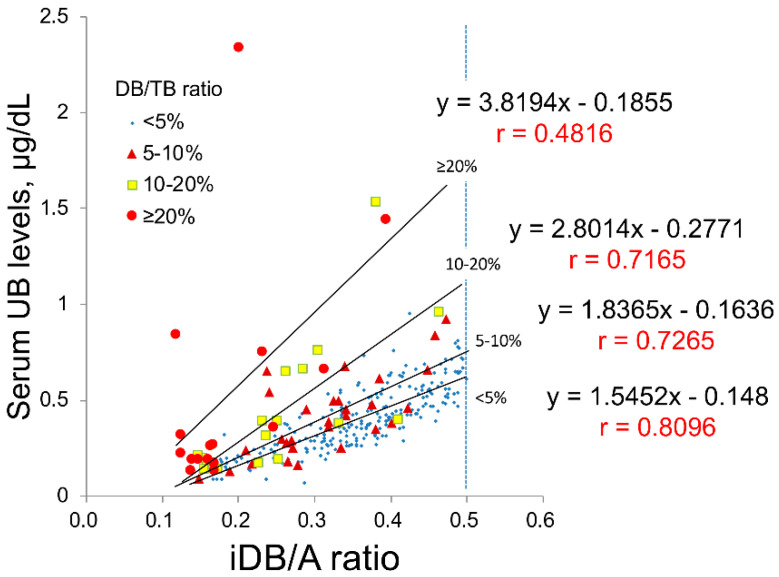
Correlations between the iDB/A ratio and UB classified by DB/TB ratio in clinical serum samples. The 301 serum samples were classified into four groups according to the DB/TB ratio for <5%, *n* = 239; 5–10%, *n* = 29; 10–20%, *n* = 17; and ≥20%, *n* = 16. A, albumin; DB, direct bilirubin; iDB, indirect bilirubin; TB, total serum bilirubin; UB, unbound bilirubin.

In this study, we collected clinical data from 345 newborn patients. To evaluate the validity of UB values, 301 samples with an indirect bilirubin/albumin (iDB/A) ratio from 0.1 to 0.5 were analyzed when the linear correlation between the UB value and iDB/A ratio was maintained (as described in Section 4). These 301 samples were then classified into four groups according to the DB/TB ratio, namely <5% (*n* = 239), 5–10% (*n* = 29), 10–20% (*n* = 17), and ≥20% (*n* = 16). The correlation (slope and correlation coefficient) between UB values and the iDB/A ratio in each group is shown in Figure 1; the slope increases and the correlation coefficient decreases with increasing DB/TB ratio.

**Figure 2:**
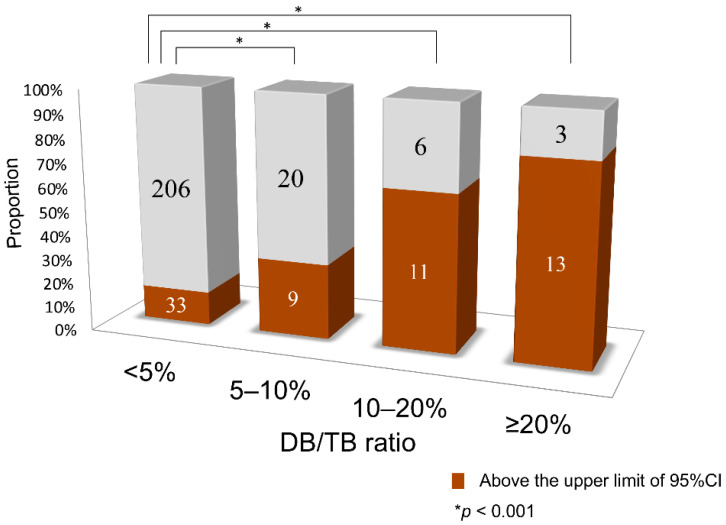
Comparison of the number of samples higher than the upper limit line of the 95% confidence interval of the DB/TB ratio <5% (outliers). CI, confidence interval; DB, direct bilirubin; TB, total serum bilirubin.

The number of samples above the line corresponding to the upper limit of the 95% confidence interval (CI) in the DB/TB <5% group (outliers) was compared between each group. In comparison to the DB/TB ratio <5% group, in which 14% of samples were above the upper limit line of the 95% CI, the other groups had a significantly higher proportion of samples above this upper limit, with 31% of the 5–10% group, 65% of the 10–20% group, and 81% of the ≥20% group (*p* < 0.001, Figure 2).

### 2.2. Study B: Impact of Serum DB Levels on Measuring Serum UB Levels Using the GOD–POD–UnaG Method

A total of 49 serum samples from 34 newborn patients were classified as having a low DB/TB ratio (<5%, *n* = 38) or a high DB/TB ratio (≥5%, *n* = 11) for analysis. The low DB/TB ratio samples were obtained from 30 newborn patients (median gestational age: 38 weeks and median birth weight: 2707 g), with a median age at sampling of five days (1–19 days), median TB level of 13.0 mg/dL (3.1–18.2 mg/dL), median DB level of 0.2 mg/dL (0.1–0.3 mg/dL), and median UB level of 0.48 µg/dL (0.04–0.87 µg/dL).

Table 1 displays the details of the 11 samples with high DB/TB ratios. These samples were obtained from four patients (one patient with trisomy 18, two patients with congenital cytomegalovirus (CMV) infection, and one patient with methylmalonic acidemia). The median TB level for these patients was 8.60 mg/dL, median DB level was 3.50 mg/dL, median iDB level was 2.60 mg/dL, median DB/TB ratio was 69.4%, and median albumin level was 3.10 g/dL. Of these 11 samples, one sample resulted in a DB/TB ratio of 5–10%, two samples with DB/TB ratio of 10–20% and eight samples with DB/TB ratio of 20% or more.

**Figure 3:**
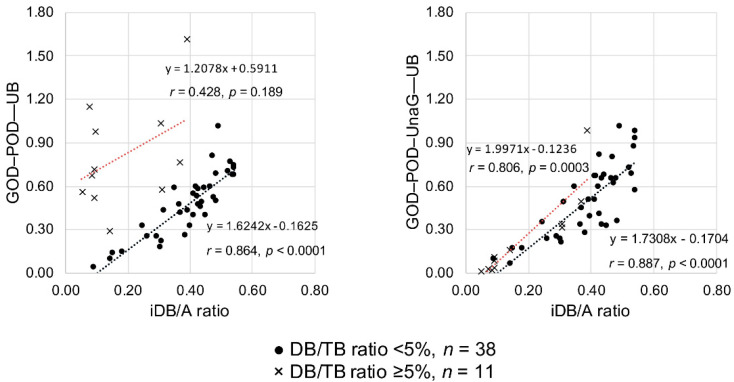
Correlations between the iDB/A ratio and serum UB values measured with the GOD–POD and GOD–POD–UnaG methods. A, albumin; DB, direct bilirubin; GOD, glucose oxidase; iDB, indirect bilirubin; POD, peroxidase; TB, total serum bilirubin; UB, unbound bilirubin; UnaG, bilirubin-inducible fluorescent protein from eel muscle.

To test the validity of UB values determined by the GOD–POD method or the GOD–POD–UnaG method, we investigated the correlation between the UB values and iDB/A ratios of low DB/TB ratio samples (*n* = 38) and high DB/TB ratio samples (*n* = 11). When the GOD–POD method was used, the 38 samples with a low DB/TB ratio showed a significant correlation (*r* = 0.864, *p* < 0.0001), whereas the 11 samples with a high DB/TB ratio did not show any correlation (*r* = 0.428, *p* = 0.189). In contrast, when the GOD–POD–UnaG method was employed, the 38 samples with a low DB/TB ratio showed a significant correlation (*r* = 0.887, *p* < 0.0001) and the 11 samples with a high DB/TB ratio also showed a significant correlation (*r* = 0.806, *p* = 0.0003) (Figure 3). More specifically, when dividing the DB/TB ratio into four groups as in Study A, applying the GOD–POD method to serum with a DB/TB ratio ≥ 5% resulted in deviation from the regression line of low DB/TB ratio serum (*n* = 38). Employing the GOD–POD–UnaG method resulted in samples plotted near the regression line of low DB/TB ratio serum (*n* = 38) regardless of the DB/TB ratio (Figure S1).

**Figure 4:**
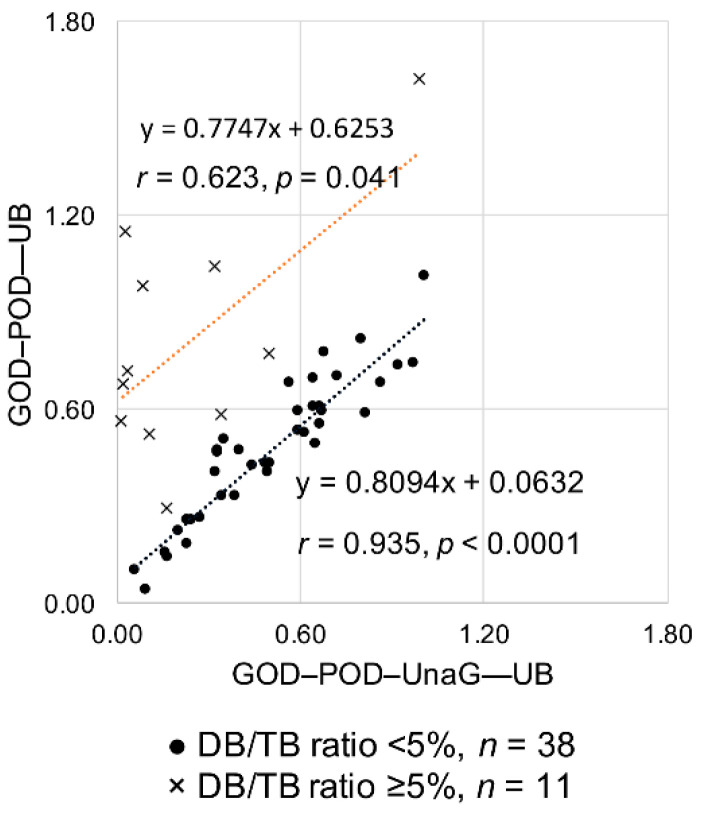
Correlation analysis of UB values determined from the GOD–POD and GOD–POD–UnaG methods. DB, direct bilirubin; GOD, glucose oxidase; POD, peroxidase; TB, total serum bilirubin; UB, unbound bilirubin; UnaG, bilirubin-inducible fluorescent protein from eel muscle.

Table 1 shows the UB values for the 11 high DB/TB ratio samples obtained from the GOD–POD and GOD–POD–UnaG methods. Despite measuring the same samples, the resulting values differed considerably between the two methods, with the UB values obtained from the GOD–POD method being significantly higher than those from the GOD–POD–UnaG method (*p* = 0.0011). For example, in Sample 1, although the DB/TB ratio was 8.2%, the UB values determined from the GOD–POD and GOD–POD–UnaG methods were quite different (1.62 and 0.99 µg/dL, respectively). We then analyzed the correlation between UB values determined from the GOD–POD and GOD–POD–UnaG methods. The 38 samples with low DB/TB ratio showed a significant correlation (*r* = 0.935, *p* < 0.0001) between the two methods, whereas the 11 samples with high DB/TB ratio resulted in lower correlation (*r* = 0.623, *p* = 0.041). The UB values determined with the GOD–POD method were higher than those determined with the GOD–POD–UnaG method (Figure 4). Figure S2 shows the graph of UB values determined from the GOD–POD and GOD–POD–UnaG methods with the DB/TB ratios classified into four groups as in Study A. Although there is no fixed trend depending on the DB/TB ratio values, sera having a DB/TB ratio of ≥ 5% often deviated from the correlation line between the two.

## 3. Discussion

In Study A, the GOD–POD method had a lower correlation with the UB value in the presence of DB with a DB/TB ratio ≥5% in newborn serum. This impact became more profound as the DB/TB ratio became larger and the number of outliers increased. It is reported that it is difficult to obtain accurate UB levels from the GOD–POD method when the DB/TB ratio is ≥10% [1]. Our findings reveal that the influence of the DB/TB ratio indeed occurs at ratios lower than 10% (and at least 5%). In Study B, we validated the GOD–POD–UnaG method that we developed for comparison with the established GOD–POD method using clinically-obtained neonatal sera. With low DB/TB ratio (< 5%) sera, the UB values from the GOD–POD–UnaG method were similar to those of the GOD–POD method. Notably, when using the GOD–POD–UnaG method, we demonstrated for the first time that even a high DB/TB ratio (≥5%) of serum does not affect the UB value.

Due to the development of perinatal medical care in recent years, severely ill infants (e.g., extremely preterm infants and infants with gastrointestinal anomalies or chromosomal disorders) can now survive. Neonatologists are encountering an increase in opportunities to treat and care for newborn patients exhibiting high DB/TB ratios in NICUs [8]. These newborn patients are more likely to develop hypoalbuminemia, with many possibilities to use drugs that alter albumin binding (e.g., antimicrobial drugs, fat formulations, and indomethacin), which consequently affect bilirubin binding to albumin to result in hyper-unbound bilirubinemia [1]. Therefore, accurate UB measurements have become paramount for such patients for a favorable prognosis. In infants with conjugated hyperbilirubinemia, there is an urgent need to develop a methodology that can measure UB levels regardless of the presence of DB.

Analysis of the correlation between UB and the iDB/A ratio was limited to serum with an iDB/A ratio <0.5; the relationship between the two certainly remains linear when the iDB/A ratio <0.5 [11], i.e., the equilibrium state of the first binding site of albumin is established. In fact, when the ratio was ≥0.6, the linear relationship between UB and the iDB/A ratio may not hold due to an equilibrium condition associated with the second binding site of albumin. As this study concerned the influence of DB in the GOD–POD method, we limited the analysis to serum samples with an iDB/A ratio <0.5 so that the analysis was simpler and easier to understand.

**Figure 5:**
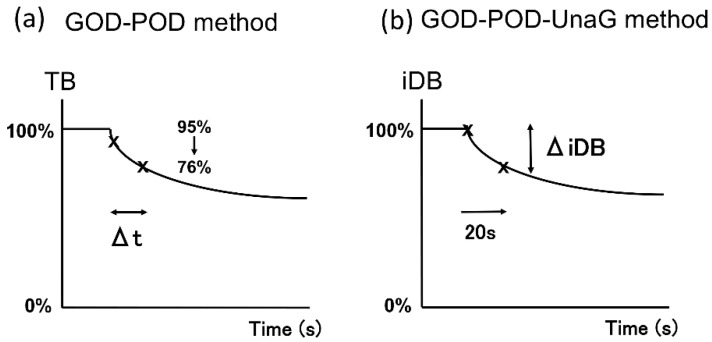
Calculation method of UB using estimated initial velocity of the GOD–POD and GOD–POD–UnaG methods. (**a**) In the GOD–POD method, the UB value is calculated by estimating the initial velocity based on the time taken for TB to reduce by 20% (Δt). (**b**) In the GOD–POD–UnaG method, the UB value is calculated based on the rate of reduction of iDB (ΔiDB) over 20 s. GOD, glucose oxidase; iDB, indirect bilirubin; POD, peroxidase; UB, unbound bilirubin; UnaG, bilirubin-inducible fluorescent protein from eel muscle.

The UB-Analyzer (UA-2) that uses the GOD–POD method can measure serum TB and UB levels [7]. UB is rapidly oxidized to colorless compounds by POD in the presence of hydrogen peroxide derived from glucose by mediation of GOD. First, TB levels are determined by direct absorbance measurement at 460 nm. Next, under experimental conditions where bilirubin oxidation follows first-order kinetics, the rate constant is determined by measuring the oxidation velocity of bilirubin in the absence of albumin. The initial velocity is estimated from the time required for a 20% decrease in concentration from the initial TB concentration. UB is calculated from the initial velocity of bilirubin degradation and the ratio of the POD concentration to that in the standard assay of the albumin-free bilirubin solution [1,6,7] (Figure 5a). As UnaG is a protein characterized by iDB concentration-dependent fluorescence [9,10], the GOD–POD–UnaG method involves the division of the serum sample into two samples to calculate the difference in TB level initially and after 20 s of POD reaction (TB reduction rate). Therefore, it is necessary to stop the POD reaction after 20 s, which can be accomplished by adding ascorbic acid (patent registration number: 6716108) (Figure 5b). As a result, we were able to establish a GOD–POD–UnaG method with a fixed reaction time (20 s), which is beneficial for the development of an automated measuring device that is currently under development in our laboratories.

Because UnaG emits very intense fluorescence and accurate measurements are hindered by an internal shielding effect when a high concentration of iDB is present, we diluted the solutions 800-fold. However, if we consider the equilibrium relationship between bilirubin and albumin, it is important to use a more concentrated solution for the UB measurement requiring the reaction with POD [12]; an 800-fold dilution for the reaction with POD would result in inaccurate UB levels. Therefore, a key feature of this GOD–POD–UnaG method is the use of a two-step dilution process, whereby the POD reaction proceeds first with a 51-fold dilution in the same manner as the GOD–POD method, and then a subsequent 800-fold dilution is used for the UnaG fluorescence measurement.

In clinical practice, a newborn patient with a high serum DB/TB ratio may require phototherapy and exchange transfusion for treatment, which is administered based on the UB values measured by the GOD–POD method; however, if judged by the results from the GOD–POD–UnaG method, this patient does not need such treatments. Out of the 11 high DB/TB ratio serum samples in Table 1, three serum samples (Samples 1, 4, and 11) had UB values ≥1.0 from the GOD–POD method, which indicates the need for exchange transfusion according to the 1992 Kobe University Treatment Criteria [13,14]. However, the GOD–POD–UnaG results for these samples indicated that not only exchange transfusion but also phototherapy would be unnecessary for the patients of serum samples 4 and 11. The results from the GOD–POD–UnaG method for serum sample 1 indicate criteria for exchange transfusion [13,14], even when taking the influence of DB into consideration. Therefore, the patient with serum sample 1 could be clearly diagnosed with serious unconjugated hyperbilirubinemia. Furthermore, while the results from the GOD–POD method for three other samples (samples 6, 7, and 8) indicated the need for phototherapy (UB ≥0.6) for these patients based on the 1992 Kobe University Treatment Criteria [13,14], the results from the GOD–POD–UnaG method indicate that phototherapy is unnecessary. Indeed, a clinical issue in Japan is the overestimation of UB values from the GOD–POD method that can lead to the overtreatment of hyperbilirubinemia. As there were patients with serious UB levels even after removing the impact of DB, it is desirable to confirm UB values of high DB/TB ratio serum samples with the GOD–POD–UnaG method.

A limitation of this study was the number of cases examined. However, we were able to show clearly from both clinical and fundamental science perspectives that the GOD–POD method affects the UB levels when the DB/TB ratio in patient serum is ≥5%. As the GOD–POD–UnaG method in this study involved manual labor and the use of expensive microplate readers, we were unable to examine numerous serum samples. Going forward, an automated device needs to be developed to perform GOD–POD–UnaG measurements with a larger number of newborn sera to verify these results and provide a clinically feasible alternative to the GOD–POD method. Finally, because other novel UB measurement methods using a fluorescence sensor or potentiometric sensor are developed [15,16], further studies are needed to compare the UB levels between their methods and our GOD–POD–UnaG method.

## 4. Materials and Methods

### 4.1. Study A

#### 4.1.1. Setting and Study Design

We conducted a multicenter retrospective study using the clinical data of serum TB and DB values in newborns admitted at the NICU at Kobe University Hospital, Kakogawa City Hospital, and Hyogo Prefectural Kobe Children’s Hospital, Japan, from 2011 to 2014. The study protocol was approved by a central institutional review board at Kobe University Hospital (approval no. 1825, date 27 October 2015). Formal written informed consent was not required due to the retrospective nature of the study, which used anonymized data generated from our regular practice. This study protocol had an announcement on our website.

#### 4.1.2. Subjects

We retrospectively analyzed the blood test values of newborn patients admitted for jaundice management that exhibited TB levels ≥5.0 mg/dL (one test value per patient). A total of 345 patients, including 270 patients with DB <0.5 mg/dL and 75 patients with DB ≥0.5 mg/dL were analyzed.

#### 4.1.3. Measurement Methods

Serum UB levels were measured using the UB-Analyzer (UA-2). TB and DB measurements were performed using the bilirubin oxidase method (IatroLQ T-bil and IatroLQ D-bil kits (Unitika Co., Okazaki, Japan) or Nescoat VL T-bil and Nescoat VL D-bil kits (Alfresa, Osaka, Japan)) or the vanadic acid oxidation method (Total Bilirubin E-HA Test and Direct Bilirubin E-HA Test (Wako Co., Osaka, Japan)). The IatroLQ D-bil kit detects only conjugated bilirubin as DB. Meanwhile, the Nescoat VL D-bil kit and Direct Bilirubin E-HA Test detect conjugated bilirubin and 𝛅-bilirubin as DB. Serum iDB concentrations were calculated using the formula (iDB) = (TB) − (DB) with 1 mg/dL = 17.1 μM. Serum albumin was measured using a modified bromocresol purple method (Kainos Laboratories, Inc., Tokyo, Japan).

#### 4.1.4. Study Methods

When the serum DB level can be regarded as almost zero and the TB/albumin (TB/A) molar ratio is between 0.1 and 0.5, the TB/A ratio is linearly correlated with serum UB [11]. In the present study, to work with sera with a high level of DB, we investigated the correlation between iDB/albumin (iDB/A) molar ratio and UB values. The correlation (correlation coefficient and the slope) between UB values and the iDB/A ratio was compared by classifying patients into four groups of DB/TB ratios (i.e., <5%, 5–10%, 10–20%, and ≥20%). In addition, the 95% CI was set using the DB/TB ratio <5% group, with the number of samples above the upper limit of the 95% CI (outliers) compared between the groups. Statistical analyses were performed with the Chi-square test, or Fisher’s exact test as appropriate.

### 4.2. Study B

#### 4.2.1. Setting

For study B, blood samples were obtained from newborns for routine laboratory tests for a variety of medical reasons at the NICU in Kobe University Hospital. Residual blood samples after performing regular laboratory tests were immediately centrifuged at 3000× *g* rpm for 10 min in the dark, and then the sera were stored at −20 °C in the dark until used. UnaG was provided by the Brain Science Institute, RIKEN, Japan [9]. The study protocol was approved by the ethical committee of Kobe University Graduate School of Medicine (approval no. 1618, date 20 August 2014). Informed consent was obtained from parents of newborns prior to blood sample collection. The methods were carried out in accordance with the approved guidelines.

#### 4.2.2. GOD–POD Method Protocol

The UB-Analyzer (UA-2) was used in accordance with the recommended manufacturer instructions. Briefly, 1000 μL of phosphate buffered saline (PBS) with glucose was first mixed with 20 μL of artificial iDB solution or sera (51-fold dilution). The reaction was then initiated with the addition of 25 μL GOD–POD solution. The initial velocity of total bilirubin oxidation was monitored by absorbance spectroscopy, and then automatically calculated as the UB value [7].

#### 4.2.3. GOD–POD–UnaG Method Protocol

**Figure 6:**
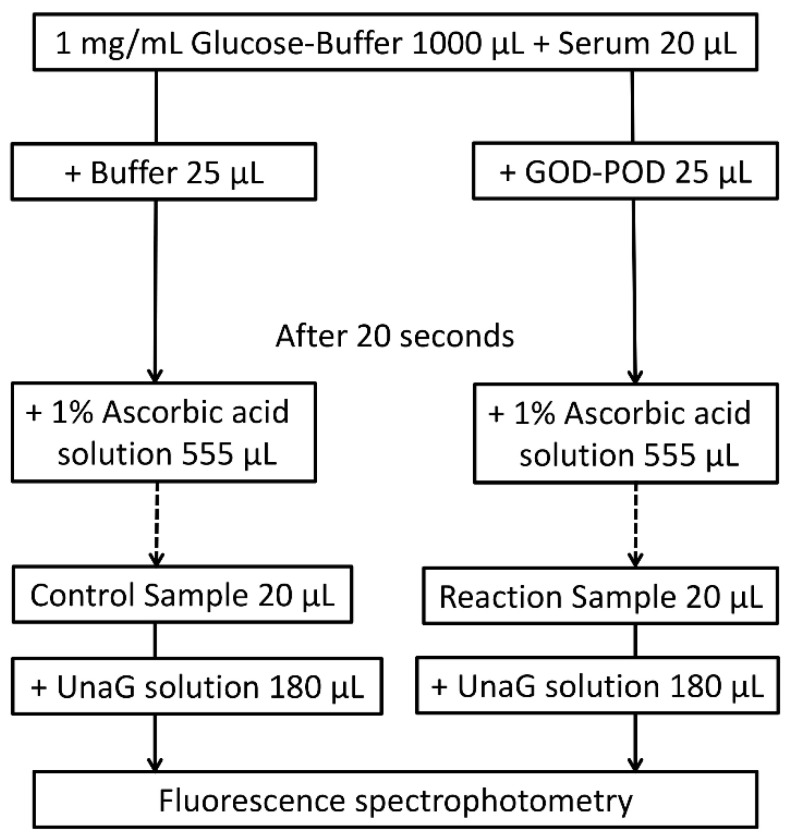
GOD–POD–UnaG method protocol. GOD, glucose oxidase; POD, peroxidase; UnaG, bilirubin-inducible fluorescent protein from eel muscle.

According to the original protocol for the UB-Analyzer using the GOD–POD method, 1000 μL of PBS with glucose was mixed with 20 μL of artificial iDB solution or sera (51-fold dilution). The reaction was then initiated with the addition of 25 μL GOD–POD solution (reaction) or PBS (control) at 37 °C. After 20 s, the reactions were quenched with 555 μL of a 1.0% ascorbic acid solution (1600 μL total volume). We confirmed that the concentration of ascorbic acid has no impact on the measurement of the iDB level (Table S1). A mixture of 200 µL containing 20 µL of the above solution and 180 µL of a UnaG solution (400 pmol) was prepared (800-fold dilution). The concentration of iDB in the reaction and control mixtures was measured from UnaG fluorescence using a microplate reader (SH-9000; Corona Electric Co., Hitachinaka, Japan) at 37 °C with fluorescence filters for excitation and emission wavelengths of 498 and 527 nm, respectively (Figure 6) [9,10]. Using the formula ΔiDB/ΔTime = K × (POD) × (UB), K was determined by decreasing the iDB levels in the artificial iDB solution (ΔiDB) (supplied by Arrows Co. Ltd., Osaka, Japan) during the POD reaction. Serum UB levels were then calculated from the difference in iDB between the reaction and control mixture solutions to calculate K as the initial velocity of iDB.

#### 4.2.4. Statistical Analysis

Statistical analyses were performed with the Mann–Whitney nonparametric rank test to compare the two independent datasets, which are expressed as the median (range) (Table 1). Regression analysis was performed to linearly compare UB values using either the GOD–POD–UnaG or GOD–POD method with the iDB/A ratio and regression equations. Correlation coefficients (*r*) were calculated using JMP 13.0.0 (SAS Institute, Cary, NC, USA). Correlations and differences were deemed statistically significant when *p* < 0.05.

## 5. Conclusions

We clearly demonstrated that UB levels using the GOD–POD method can be affected when the DB/TB ratio >5%, suggesting that DB should be easily oxidized by POD and lead to an overestimation of the UB value. Importantly, we developed the GOD–POD–UnaG method as a novel UB measurement method. This method can measure the UB levels in newborn sera, regardless of the presence of DB, highlighting an attractive alternative method to the conventional GOD–POD assay, especially for the prognosis of newborns with high DB serum.

## 6. Patents

The GOD–POD–UnaG method was registered with the international patent office on 12 June 2020 (registration number: 6716108).

## Figures and Tables

**Table 1:** Characteristics of high DB/TB ratio samples.

Sample Number	Case Number	TB (mg/dL)	DB (mg/dL)	iDB (mg/dL)	DB/TB (%)	Alb (g/dL)	UB (µg/dL)	
							GOD–POD	GOD–POD–UnaG
#1	a	14.7	1.2	13.5	8.2	4.1	1.62	0.99
#2	a	9.1	1.0	8.1	11.0	2.6	0.77	0.50
#3	a	9.6	1.5	8.1	15.6	3.1	0.58	0.34
#4	a	9.1	2.1	7.0	23.1	2.7	1.04	0.32
#5	b	5	1.3	3.7	26.0	1.9	0.29	0.16
#6	c	7.2	5.0	2.2	69.4	3.1	0.68	0.02
#7	c	7.9	5.5	2.4	69.6	3.1	0.72	0.04
#8	c	8.6	6.0	2.6	69.8	3.3	0.98	0.09
#9	c	8.7	6.1	2.6	70.1	3.4	0.52	0.11
#10	b	4.9	3.5	1.4	71.4	2.8	0.56	0.02
#11	d	8.0	6.0	2.0	75.0	3.2	1.15	0.03
Median		8.60	3.50	2.60	69.44	3.10	0.72	0.11
							*p* = 0.0011	

Case. a: 38 weeks of gestational age at birth, 744 g of birth weight, trisomy 18. b: 39 weeks of gestational age at birth, 2526 g of birth weight, congenital cytomegalovirus infection. c: 39 weeks of gestational age at birth, 2054 g of birth weight, congenital cytomegalovirus infection. d: 38 weeks of gestational age at birth, 2362 g of birth weight, methylmalonic acidemia. Alb, albumin; BW, birth weight; DB, direct bilirubin; GA, gestational age; GOD, glucose oxidase; iDB, indirect bilirubin; POD, peroxidase, TB, total bilirubin; UB, unbound bilirubin; UnaG, bilirubin-inducible fluorescent protein from eel muscle.

## Supplementary Materials

Supplementary Materials can be found at https://www.mdpi.com/1422-0067/21/18/6778/s1.

## Abbreviations

iDB	Indirect bilirubin (serum unconjugated bilirubin)
UB	Unbound bilirubin (albumin free bilirubin)
GOD	Glucose oxidase
POD	Peroxidase
GOD–POD	Glucose oxidase–peroxidase
DB	Direct bilirubin (conjugated bilirubin)
NICU	Neonatal intensive care unit
UnaG	Bilirubin-inducible fluorescent protein from eel muscle
TB	Total serum bilirubin
iDB/A	Indirect bilirubin/albumin
CI	Confidence interval
CMV	Cytomegalovirus
TB/A	Total serum bilirubin/albumin
PBS	Phosphate buffered saline
