# Supplementary material for: A Novel Method for Measuring Serum Unbound Bilirubin Levels Using Glucose Oxidase–Peroxidase and Bilirubin-Inducible Fluorescent Protein (UnaG): No Influence of Direct Bilirubin
Source: Int J Mol Sci. 2020 Sep 16;21(18):6778. doi: 10.3390/ijms21186778 (PMC7555467; doi:10.3390/ijms21186778)
Supplement: Supplementary file 1 [file ijms-21-06778-s001.zip › ijms-21-06778-s001/Table S1.pdf]

Table S1. Effect of ascorbic acid in serum on unconjugated bilirubin (indirect bilirubin) measurments by UnaG mthod.

Sample	Ascorbic acid (mg/mL)	iDB levels (mg/dL)
(a)	1000 (100%)	15.7
	100 (10%)	15.6
	10 (1%)	15.3
	1 (0.1%)	15.3
	0 (0%)	15.4
(b)	1000 (100%)	8.2
	100 (10%)	8.1
	10 (1%)	8.3
	1 (0.1%)	8.0
	0 (0%)	8.2

Serum samples (a) and (b) contained an iDB of 16.9 and 7.8 mg/dL, respectively, as determined by the bilirubin oxidase method. See in Section 4.1.3.

UnaG method was carried out based on the reference #10.

iDB, indirect bilirubin; UnaG, bilirubin-inducible fluorescent protein from eel muscle.
