# Supplementary figures and images for: A Novel Method for Measuring Serum Unbound Bilirubin Levels Using Glucose Oxidase–Peroxidase and Bilirubin-Inducible Fluorescent Protein (UnaG): No Influence of Direct Bilirubin
Source: Int J Mol Sci. 2020 Sep 16;21(18):6778. doi: 10.3390/ijms21186778 (PMC7555467; doi:10.3390/ijms21186778)

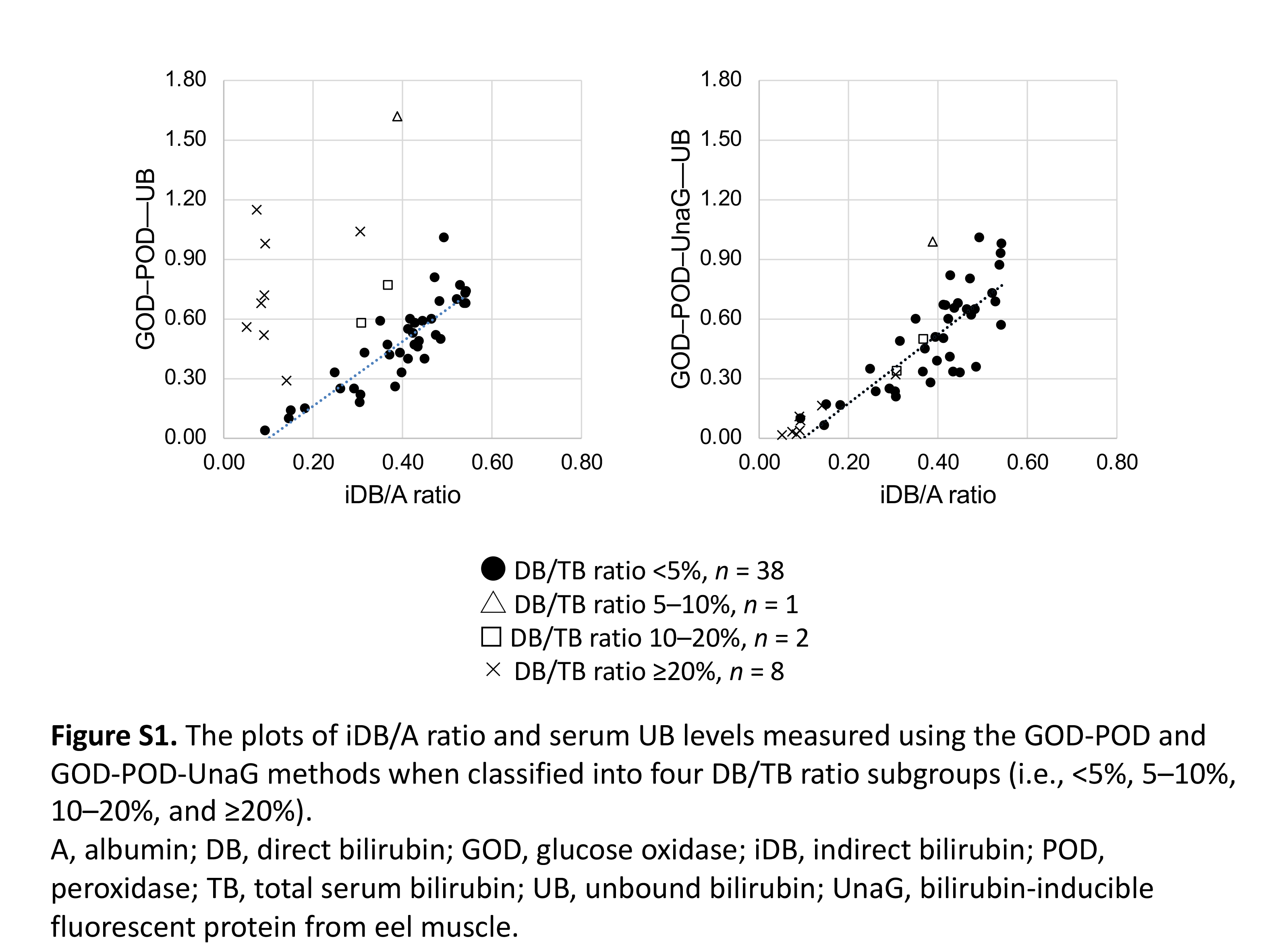

Supplement: Supplementary file 1 [file ijms-21-06778-s001.zip › ijms-21-06778-s001/Figure_S1.tif]

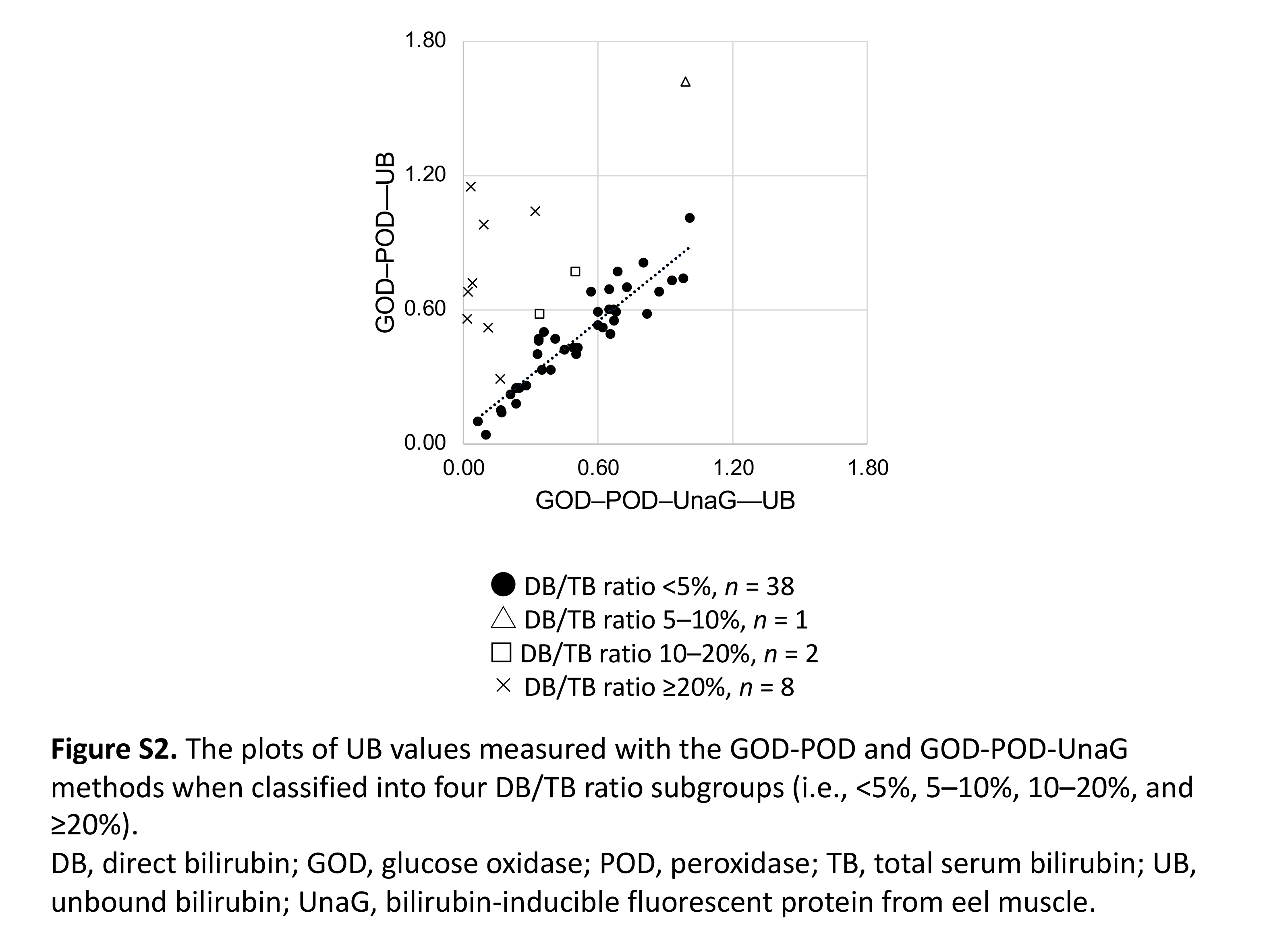

Supplement: Supplementary file 1 [file ijms-21-06778-s001.zip › ijms-21-06778-s001/Figure_S2.tif]
